# Valedictory to the Graduates of Rush Medical College, February 5th, 1862

**Published:** 1862-02

**Authors:** 


VALEDICTORY to the Graduates of Rush Medical College, February 5th, 1862. By J. W. FREER, M. D., Professor of Physiology and Surgical Anatomy.

We acknowledge the receipt of a neatly printed copy of Prof. FREER's *Address*, delivered as above.

About all has been, that can be said on such occasions, and he must be a genius who shall advance something new. But words of advice so earnest, sound and sensible as these, are always in season; and if they should lack the "unwholesome charm of novelty," are not the less valuable or acceptable.

The following points are extremely well put:—

The strong point of the charlatan and quack is in social chicanery and blaring demonstrations of what he has done and can do; the strength of the educated medical man is in his knowledge, gathered by such assistants as these—interrogating nature and demanding possession of its arcana. There is more real power in the microscope and the test tube than in the whole army of empirics, from the first quack who said: "Ye shall not surely die," to the last and meanest follower of Hahemann, who repeats to his deluded dupes a similar promise. * * * *

You are scrupulously to avoid, at all times and on all occasions, any and all disreputable professional associations; but grapple to your breasts, with hooks of steel, every upright, intelligent and high-minded physician—for in union there is strength. And alas! it is for want of this union—particularly in our own country—that our noble and beneficent calling has been measurably shorn of its sacredness—has been drabbled and draggled in the dust by every aspiring knave—until (I

blush to say it) the public have come to look upon pretension as the measure of qualification, and a gaudy tin sign, with staring hieroglyphics, as equal to a diploma from the most renowned institution of the world! Look to it, then, that you guard sacredly the honor and interests of your acknowledged professional brethren, for in doing thus, you will compel public respect, by convincing them that we are not a band of wranglers, but on the contrary harmoniously engaged in the completer development of principles embracing in their scope the highest temporal interests of the human race. * * *

The charge is frequently made that medicine, as a science, has been undergoing constant mutations. If by this, it is intended that it is a progressive science, we admit it freely; it is the glory of the profession. But, if by this, it is intended that it has been a history of contradictions—we repel it as begotten either of calumny or ignorance. Methods of accomplishing results have varied and will continue to vary, as men and times, seasons and surroundings change.

The objects sought by Hippocrates, we still seek to-day, and will continue to seek during all coming time. Powers and Turner may, and probably do adopt different methods of giving shape to their glowing ideals, from those adopted by Praxiteles and Appelles; but the same eternal principles vitalize all their creations. You may rest assured that were Hippocrates now living, he would be the disciple of no pathy or—ism—even to secure admission to the luxurious apartments of the hysterical or dyspeptic votaries of infinitesimal triturations.

